# Engineered nanomaterials: toward effective safety management in research laboratories

**DOI:** 10.1186/s12951-016-0169-x

**Published:** 2016-03-15

**Authors:** Amela Groso, Alke Petri-Fink, Barbara Rothen-Rutishauser, Heinrich Hofmann, Thierry Meyer

**Affiliations:** Occupational Safety and Health, School of Basic Sciences, Ecole Polytéchnique Fédérale de Lausanne, Lausanne, Switzerland; Group of Chemical and Physical Safety, Ecole Polytéchnique Fédérale de Lausanne, Lausanne, Switzerland; BioNanomaterials, Adolphe Merkle Institute, University of Fribourg, Ch. des Verdiers 4, 1700 Fribourg, Switzerland; Chemistry Department, University of Fribourg, Ch. Du Musée 9, 1700 Fribourg, Switzerland; Powder Technology Laboratory, Ecole Polytéchnique Fédérale de Lausanne, Lausanne, Switzerland

**Keywords:** Risk assessment, Occupational health and safety

## Abstract

**Background:**

It is still unknown which types of nanomaterials and associated doses represent an actual danger to humans and environment. Meanwhile, there is consensus on applying the precautionary principle to these novel materials until more information is available. To deal with the rapid evolution of research, including the fast turnover of collaborators, a user-friendly and easy-to-apply risk assessment tool offering adequate preventive and protective measures has to be provided.

**Results:**

Based on new information concerning the hazards of engineered nanomaterials, we improved a previously developed risk assessment tool by following a simple scheme to gain in efficiency. In the first step, using a logical decision tree, one of the three hazard levels, from H1 to H3, is assigned to the nanomaterial. Using a combination of decision trees and matrices, the second step links the hazard with the emission and exposure potential to assign one of the three nanorisk levels (Nano 3 highest risk; Nano 1 lowest risk) to the activity. These operations are repeated at each process step, leading to the laboratory classification. The third step provides detailed preventive and protective measures for the determined level of nanorisk.

**Conclusions:**

We developed an adapted simple and intuitive method for nanomaterial risk management in research laboratories. It allows classifying the nanoactivities into three levels, additionally proposing concrete preventive and protective measures and associated actions. This method is a valuable tool for all the participants in nanomaterial safety. The users experience an essential learning opportunity and increase their safety awareness. Laboratory managers have a reliable tool to obtain an overview of the operations involving nanomaterials in their laboratories; this is essential, as they are responsible for the employee safety, but are sometimes unaware of the works performed. Bringing this risk to a three-band scale (like other types of risks such as biological, radiation, chemical, etc.) facilitates the management for occupational health and safety specialists. Institutes and school managers can obtain the necessary information to implement an adequate safety management system. Having an easy-to-use tool enables a dialog between all these partners, whose semantic and priorities in terms of safety are often different.

**Electronic supplementary material:**

The online version of this article (doi:10.1186/s12951-016-0169-x) contains supplementary material, which is available to authorized users.

## Background

According to the ISO 31000:2009 norm [1], risk is often expressed in terms of a combination of the consequences of an event and the associated likelihood of occurrence. Risk evaluation and mitigation are major concerns for occupational health and safety (OHS) specialists working in organization for economic cooperation and development (OECD) countries, in which employers are obliged to evaluate all the risks their workers may face [2].

Evaluating the risk of a chemical substance to cause harm involves the compilation of accurate detailed information from hazard pictograms, or labels, and material safety data sheets (SDS), based, for instance, on the new regulation system of classification and labeling, the globally harmonized system (GHS) [3]. Estimating the likelihood of occurrence requires a study of the exposure: processes and procedures, quantities handled, duration and frequency of operation, etc. [4].

Engineered nanomaterials (ENMs) often demonstrate properties that differ from the properties of the same material in the bulk form, providing opportunities for new applications [5]. The definition of nanomaterials used herein is that given by the European Commission (EC) in its 2011 report [6]. ENMs are materials intentionally produced at the workplace via chemical or physical processes.

The overall human health risk assessment concepts for chemicals appear to be applicable to nanomaterials, although adaptations may be needed for individual protocols [7]. The occupational exposure limit (OEL) defines the upper limit of the acceptable concentration of a hazardous substance at the workplace. Currently, there are no specific regulatory OELs established for ENMs. Recommended OELs have been developed for certain materials such as carbon nanotubes (CNTs) [8] and nanosized titania (nTiO_2_) [9]. Given the current state of knowledge on ENMs, it is likely that several years will be needed before we will precisely know which types of ENMs and associated doses will represent a real danger to humans and the surrounding environment [4].

In the meantime, there is a consensus on applying the precautionary principle to these novel materials until more information is available. Recommendations on safe working with ENMs have been developed in the past decade by government agencies and occupational health organizations [10–14]. Some new tools, as those based on control banding [4, 15–18] have been developed for managing the risk of ENMs in an alternative way. They use the generally accepted risk paradigm, in which risk is a function of the severity of the impact and the anticipated probability of that impact (exposure) [19].

These tools are risk-assessment approaches in the context of uncertainty regarding hazards of ENM, and in the absence of occupational exposure limits and recommendations for quantitative exposure measurements. As such they are very useful as they can provide an alternative risk assessment and risk management process, by grouping occupational settings in categories presenting similarities of hazards and/or exposure, while incorporating professional judgment and monitoring [16]. It is difficult to evaluate the performance of the approaches until now and more development is expected in their modification, adjustment and validation [19]. Uncertainty and a precautionary approach seem to result in a rather conservative allocation of hazard bands, leading to high levels of estimated risk requiring high protection [19].

We could not easily apply the existing methods to manage the ENM risks in academic research since this environment is highly versatile, has large number of laboratories, staff with very high level of education, but fast turnover and large cohorts of inexperienced people [2]. Additionally, the level of detail of the provided protective and preventive measures was not sufficient in the above-cited methods, as they give basic advices or indicate to seek for a specialist [4].

There was an urgent need for a simple and easy to use tool (not only for safety specialists but for researchers as well) that can rapidly evolve and provide preventive and protective measures for a complete life cycle of an activity involving ENMs. Indeed, research topics in the field of safety in academia are recently prioritized. Due to the occurrence of numerous accidents investigated by the U.S. Chemical Safety and Hazard Investigation Board, ABET, the U.S. Accreditation Board of Engineering and Technology, has modified recently the chemical engineering curriculum requirements. Starting in 2012 the student should obtain sufficient knowledge and know-how to operate chemical process equipment not only in a technical sense but also in conformance with health, safety, and environmental regulation [20]. As laboratory safety is not simply a matter of materials and equipment but also of processes and behaviors, a crucial component of chemical education at every level is to nurture basic attitudes and habits of prudent behavior so that safety is a valued and inseparable part of all laboratory activities [21].

We previously developed a methodology for handling ENM safety in a research environment [22]. Even though the tool was essentially based on the exposure potential estimate only, it has demonstrated its usefulness; a first screening of the laboratory activities was made, allowing further actions in protecting co-workers, especially where significant exposure potential was present.

As new facts are available regarding ENM hazards, and therefore more differentiation in determining the potential hazard level is possible, we present herein an improved control banding methodology to manage the safety of ENMs in research environments.

## Results

**Fig. 1:**
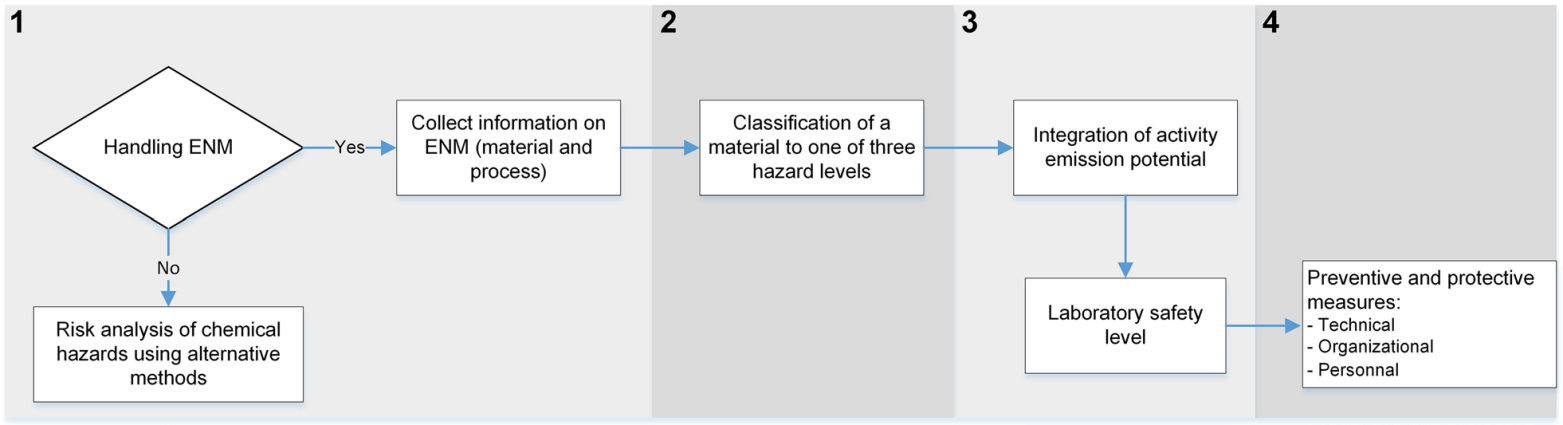
Flow chart of the process for nanolaboratory classification. Schematic presentation of the procedure for managing occupational safety and health in laboratories producing and using engineered nanomaterials

To propose a user friendly method for the determination of precautionary risk levels of an activity involving the work with ENMs, all the questions the user should answer are given in the form of schematic decision trees, with a choice of possible ‘Yes’, ‘No’, and ‘I do not know’ answers. The structure of the procedure is presented in Fig. 1 and its details and rationales are presented below.

### Eligibility of the method and information collection

If the activity concerns ENMs as defined by the EC recommendation [6], the user should collect available information on the ENM (e.g., SDS of the used material or its bulk material) and the process in question; if the answer is ‘No’, the user should adopt a risk analysis method for chemical hazards.

### Classification of substances into potential hazard levels

**Fig. 2:**
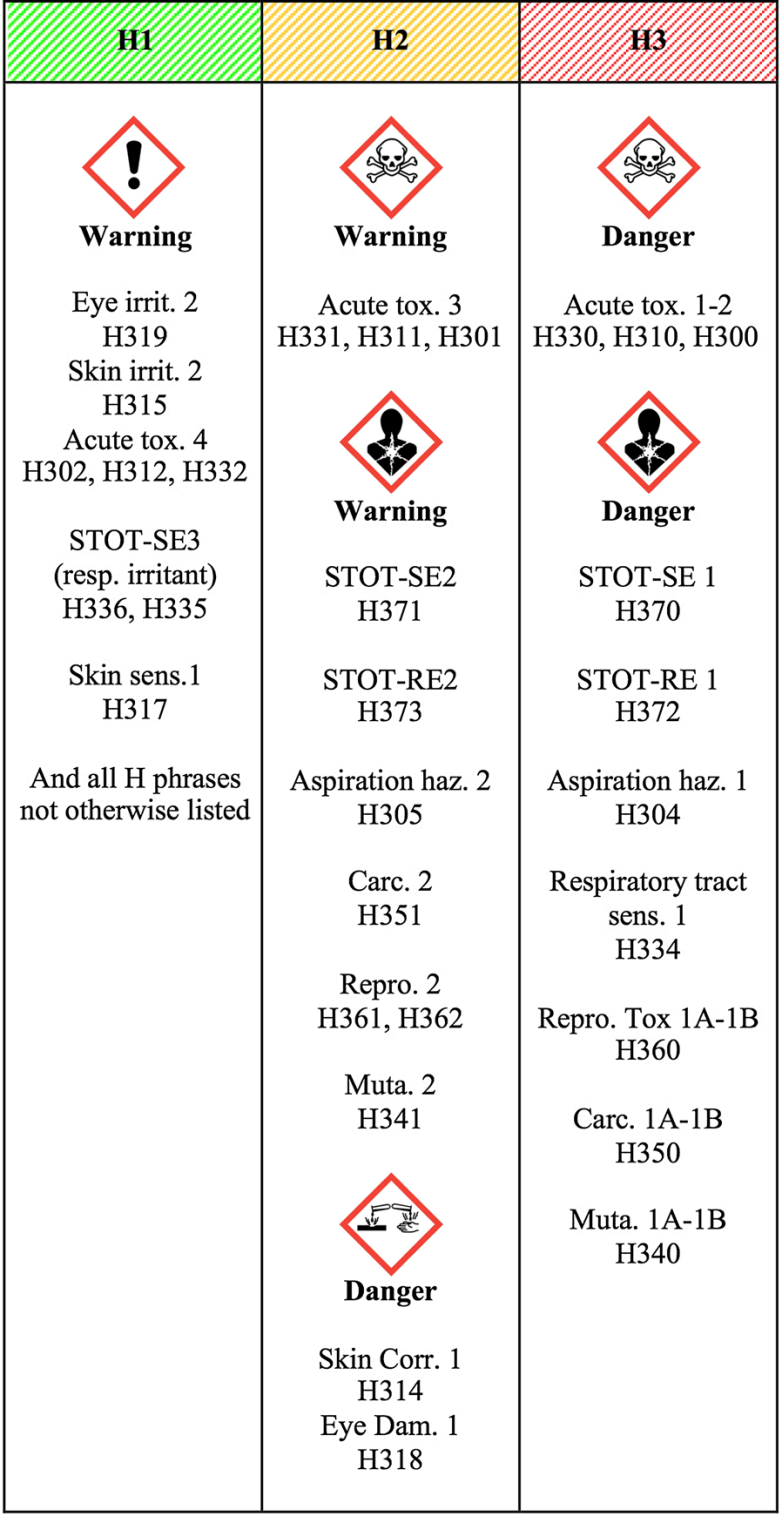
Innovative chemical substances classification into three hazard levels. Source of pictograms: Globally harmonized system of classification and labeling of chemicals (GHS), United Nations Economic Commission for Europe, 2011

The bulk material is defined as the material with the same chemical composition and crystalline phase as the ENM, but with all the external dimensions larger than 100 nm. Based on the GHS for classification and labeling of chemicals [3], we have classified the (bulk) chemical substances into three hazard levels, from H1 to H3 (see Fig. 2):

- *H1* substances presumed not to be harmful to human health (there is no report to date showing adverse effect). Effect: no significant effect to health;
- *H2* substances presumed to be harmful to human health. Effect: moderate or transient effects to health;
- *H3* substances known or presumed to have significant toxicity in humans. Effect: significant or permanent effect to health.

There are still no publications in the literature that show any confirmed effects to humans from exposure to ENM and information is only available from in vitro and in vivo animal studies. We therefore use following scale for potential hazards levels of ENM:

*H1* None of endpoints increased; *H2* Some indication that at least one of the endpoints is increased; *H3* at least one endpoint is significantly increased.

The endpoints include cytotoxicity, immunotoxicity, neurotoxicity, genotoxicity, oxidative stress, inflammation, apoptosis/necrosis; the list is not complete and it is expected that other endpoints will be determined in future studies.

**Fig. 3:**
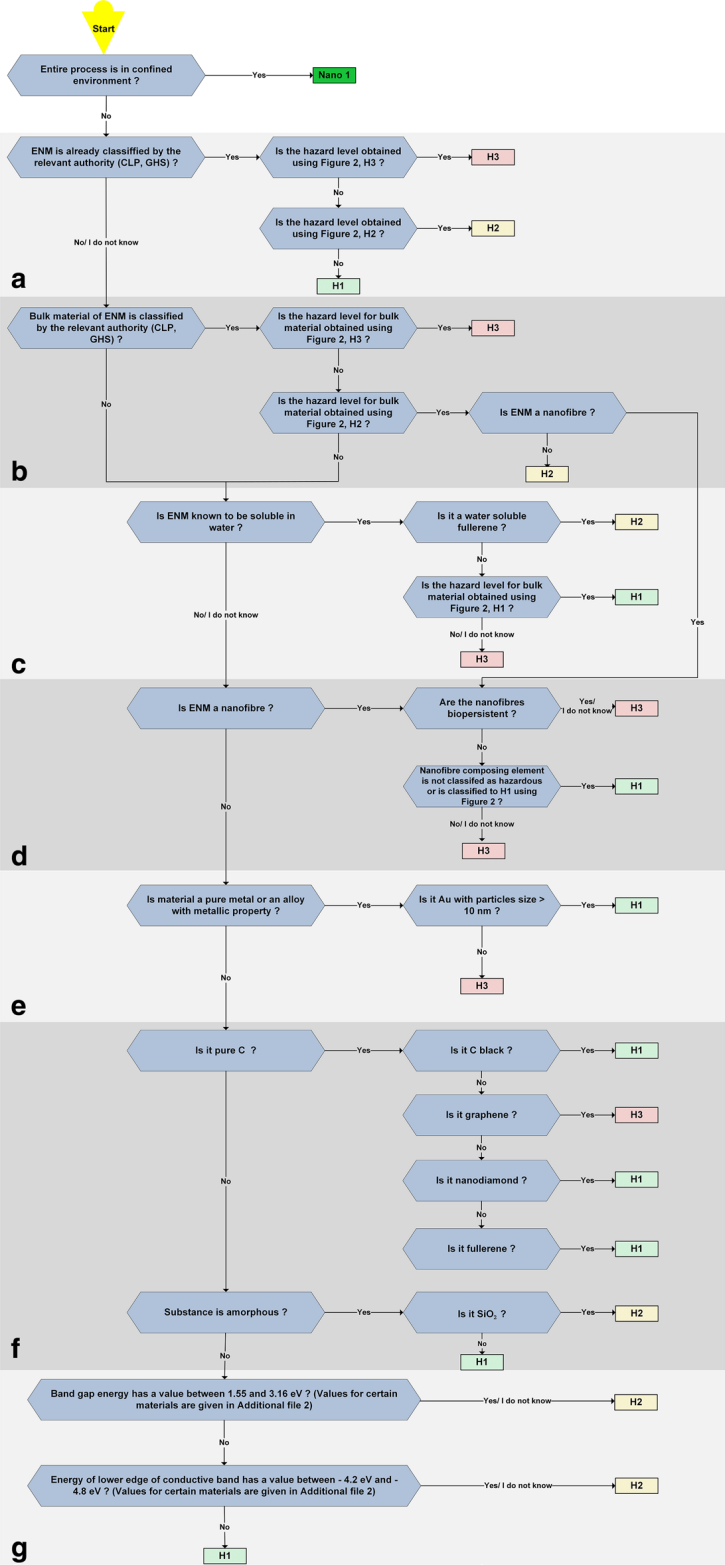
Decision tree for potential hazard level determination. Questions that should be answered by engineered nanomaterial users and producers in research environment when assigning a potential hazard level to their material. The questions are organized in seven sections: **a**, **b**…, **g**

The user shall employ the decision tree in Fig. 3 to assign a potential hazard level to the material. Each type of ENM should be analyzed separately; for hybrid particles, composed of two or more chemical elements or components, the decision tree should be applied to each element or component separately. For readability, the questions are organized in seven categories, from *a* to *g* (see Fig. 3).

*Basis for the potential hazard level estimate (Fig.* *3**).* Assuming that even hazardous substances pose no risk when confined, the first question in Fig. 3 concerns the environment, i.e., whether the process is conducted in a closed (complete process confinement) or in an open system. Examples of such confined systems are glove boxes, glove bags, and sealed chambers. If the process is not fully enclosed, different criteria on presumed or known toxicity based on the literature review are used to determine the potential hazard level.

#### Section a, Fig. 3

This part examines whether the ENM has already been studied with regard to its classification and labeling. If the answer is ‘Yes’, then the hazard of the material to human health is clearly identified. Figure 2 is used to obtain the hazard level of the ENM according to its classification. In their study, Lee et al. [23] evaluated the information provided in 97 ENM related SDSs and found that most of them did not include sufficient information on the safety of ENM such as their toxicity and physicochemical properties. As research on nanomaterials toxicity is ongoing and guidance on the preparation of SDS for ENM was made as a supplement to ISO 11014 [24], hopefully more information will be available in the future.

If the ENM is not classified, further questions are asked regarding the corresponding bulk material in section *b* of the tree.

#### Section b, Fig. 3

ENMs whose bulk material is classified as H3 or H2 (Fig. 2) are also assigned the same H level (except nanofibers that are further examined in section *d* of Fig. 3). Further criteria in the decision tree will therefore be examined only for those ENMs whose bulk material is not classified (or does not exist yet) or is classified as H1 according to Fig. 2.

#### Section c, Fig. 3

In this part, the solubility of ENMs in an aqueous environment (pH 5–7) is considered. In the context of this work, the solubility is considered to clearly differentiate between the ENM and its bulk counterpart. If the investigated ENM dissolves in water under (near) physiological conditions, the final system will be a solution composed of ions, atoms, or molecules. The toxicity of these species is already classified by the relevant authority [25].

The extent to which a species dissolves can be expressed in terms of the equilibrium constant. At the macroscopic level, a common threshold for determining the solubility is 0.1–1 g per 100 mL of water [26]. Our team has adopted a threshold of 0.1 g/L, as given in [27]. Solubility data on a large number of bulk inorganic compounds can be found in online databases [28, 29] or in standard handbooks [30].

Materials whose solubility is larger than this threshold value are considered soluble, whereas materials whose solubility is lower are considered insoluble. Notably, there is no absolute insoluble material, and the threshold applied here has been chosen knowing that small nanoparticles show an increased solubility compared to bulk materials. The ENM size is considered as the primary physicochemical property affecting the solubility; however, the ENM shape, surface area, and crystallinity have been revealed to also play major roles [31].

The solubility generally increases as the particle size decreases, as theoretically confirmed by the Ostwald–Freundlich equation [32]. The range of validity of the equation has been discussed in many manuscripts and the interested reader is referred to the respective literature [33–35].

For the described methodology to manage the ENM safety in research environments, users that handle nanoparticles should use the Ostwald–Freundlich equation to roughly estimate the influence of particle size on solubility:

1 $$S = S_{o} \exp \left( {\frac{2\gamma V}{RTr}} \right)$$

where S is the solubility (in mol kg^−1^) of spherical particles, r is the radius (in m), S_0_ is the solubility of the bulk material, V is the molecular volume (in m^3^ mol^−1^), γ is the surface tension (in J m^−2^), R is the gas constant (in J mol^−1^ K^−1^), and T is the temperature (in K). The obtained solubility S should then be checked against the above mentioned threshold value 0.1 g/L.

The solubility does not change significantly, compared to the bulk value, for particles with size between 10 and 100 nm, and the most significant enhancement in the calculated solubility is typically expected for very small particles below 10 nm. For example, using typical values for oxides (e.g., TiO_2_, anatase) the solubility of 100 nm, 10 nm, and 5 nm particles is respectively 1.2, 5.2, and 27 times larger than the bulk solubility.

As shown in Fig. 3, section **c,** three potential hazard levels are possible for soluble substances.

- Soluble fullerenes are classified as potential hazard level 2. The current information on human exposure and toxicity of fullerenes is restricted to short-term studies only. It has been shown that pristine fullerenes exhibit low toxicity, but extrapolations to various fullerene types or chronic exposure cannot be made at this point [36].
- Soluble ENM substances whose bulk material is classified as H1 are assigned the same hazard level.
- Soluble ENMs whose bulk materials are not classified as H1 or the answer is ‘I do not know’ are endorsed with the highest potential hazard level following the precautionary principle.
- Insoluble substances or substances whose solubility is not known are further examined in the section *d* of the tree.

#### Section d, Fig. 3

A nanofiber is defined as a “nano-object with two similar external dimensions in the nanoscale and the third dimension significantly larger” [37].

Fibers or high aspect ratio nanoparticles (HARN) have gained enormous interest in the field of inhalation toxicology. Previous studies on pathogenic fibers such as asbestos indicate that some parameters, such as longer length (>10–15 μm) and durability, are important in driving fiber toxicity involving fibrosis, pleural plaques, and cancer, i.e., mesothelioma, within occupational or consumer related settings [38, 39]. All findings are summarized in the so-called fiber paradigm, which describes the structure–toxicity relationship as a function of fiber length, thickness, and biopersistence (for a review, see [40]). Biopersistency might also occur for other ENM such as round shaped particles, however, it is especially a problem when the fibers cannot be engulfed by macrophages, but are released in their environment (i.e., frustrated phagocytosis [41]). This is not expected to occur for round shape particles where the majority (if not all) is taken up by the cells.

Two potential hazard levels are possible for nanofibers in our decision tree.

- Biopersistent (defined as the ability of a fiber to remain in the lung in spite of the lung physiological clearance mechanisms) nanofibers, as well as all fibers whose composing material is not classified as H1, are assigned the H3 potential hazard level.
- If a nanofiber is not biopersistent and its composing element is not classified as hazardous or is classified as H1, it is classified as potential hazard level 1.

As for the other parts of the decision tree, ‘I do not know’ answers (question on biopersistence and classification of composing materials herein) are always treated as the worst-case scenarios (potential hazard level 3).

If an insoluble ENM is not a fiber, further questions (section *e*, Fig. 3) deal with pure metals and materials with metallic properties as defined in [42].

#### Section e, Fig. 3

Among metals, gold is classified as H1 if its particle size is larger than 10 nm [43]. Gold particles with sizes below 10 nm were revealed to produce DNA damage [44] and are therefore treated as all the other metallic nanoparticles, namely, classified as H3. Regarding other noble metals like platinum or palladium we decided to classify them as H3 because either powders of these metals could have harmful health effects or not enough concerning their impact on health is known [45, 46]. This classification is based on the state of the art regarding the toxicity of metals as presented by Karlsson et al. [43].

#### Section f, Fig. 3

If a material is not a metal or a material with metallic properties, but is composed of pure C, it is treated as follows.

- Fullerenes, nanodiamonds, and carbon black are classified as H1. These three types of materials do not show toxic reactions [47, 48]. Furthermore, the occupational exposure standards and guidelines for C black are set in most of the countries [48].
- Graphene is still a relatively new material and only a few datasets regarding its toxicity exist. As such, it is classified as H3.

#### Section g, Fig. 3

The remaining categories of substances, i.e., metal oxides and semiconductors, are treated in the last part of the decision tree.

- Amorphous substances are classified as H1, except amorphous silica (SiO_2_), which is classified to potential hazard level H2. Epidemiological studies have drawn inconsistent conclusions on amorphous silica toxicity [49]. In vivo animal studies have reported a transient inflammatory response to amorphous silica [50, 51]. In vitro studies have shown that exposure of mouse alveolar macrophage cells to both, crystalline and amorphous silica results in cell death [52], the effect on other cell types is less clear.

A problem in evaluating the human effects of amorphous silica exposure is that there is usually some level of crystalline silica contaminating amorphous silica samples [53].

Registration, evaluation, authorization and restriction of chemicals (REACH) is an European regulation by which manufacturers and importers are required to gather information on the properties of their chemical substances, which will allow their safe handling, and to register the information in a central database in the European Chemicals Agency (ECHA). Silica is currently undergoing a REACH process. It is possible that we need to re-evaluate our current hazard classification when the process is completed.

- For crystalline materials, two selection criteria are used, i.e., the band gap energy and the energy of the lower edge of the conduction band. If the band gap energy is in the energy range of visible or near infrared light (from 3.16 eV at a wavelength of 400 nm to 1.55 eV at 800 nm), the excitation of the electrons in the valence band during handling under daylight conditions could be possible, increasing the photoreactivity of the particles. For example, the cytotoxicity of nTiO_2_ under light illumination was related to the generation of reactive oxygen species (ROS), a mechanism not observed in the dark [54]. If its band gap energy is in this range (from 1.55 to 3.16 eV), the ENM is classified as H2, otherwise the question on the ENM energy of the lower edge of the conduction band is examined. Zhang et al. [55] were able to show that the overlap of the conduction band energy levels with the cellular redox potential (from −4.12 eV to −4.84 eV) was strongly correlated with the ability to induce oxygen radicals, oxidative stress, and inflammation. If the energy of the lower edge of the conduction band lies between −4.2 eV and −4.8 eV, an H2 potential hazard level is assigned to the material, and an H1 hazard level otherwise. To help the user classify their materials, we have calculated (using approach as given in [56]) band gaps and energies of the lower level of the conduction bands for some common materials as functions of the particle size. The table is available in Additional file 1: Figure S1.

**Fig. 4:**
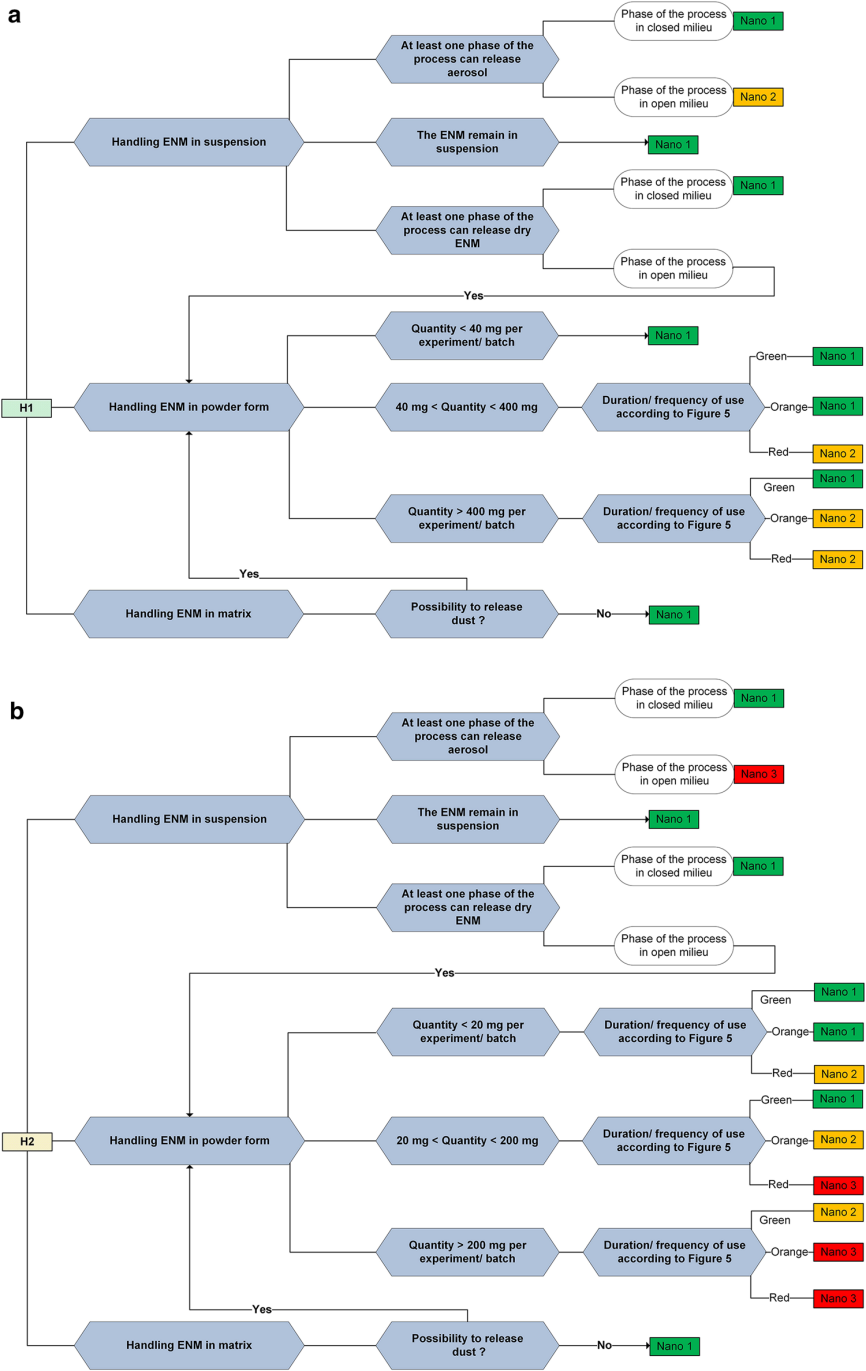
Decision tree for determining the Nano risk laboratory level. **a** Decision tree used for determining the Nano level as a function of the substance and activity emission potential for the potential hazard level H1 obtained using Fig. 3. **b** Decision tree used for determining the Nano level as a function of the substance and activity emission potential for the potential hazard level H2 obtained using Fig. 3

**Fig. 5:**
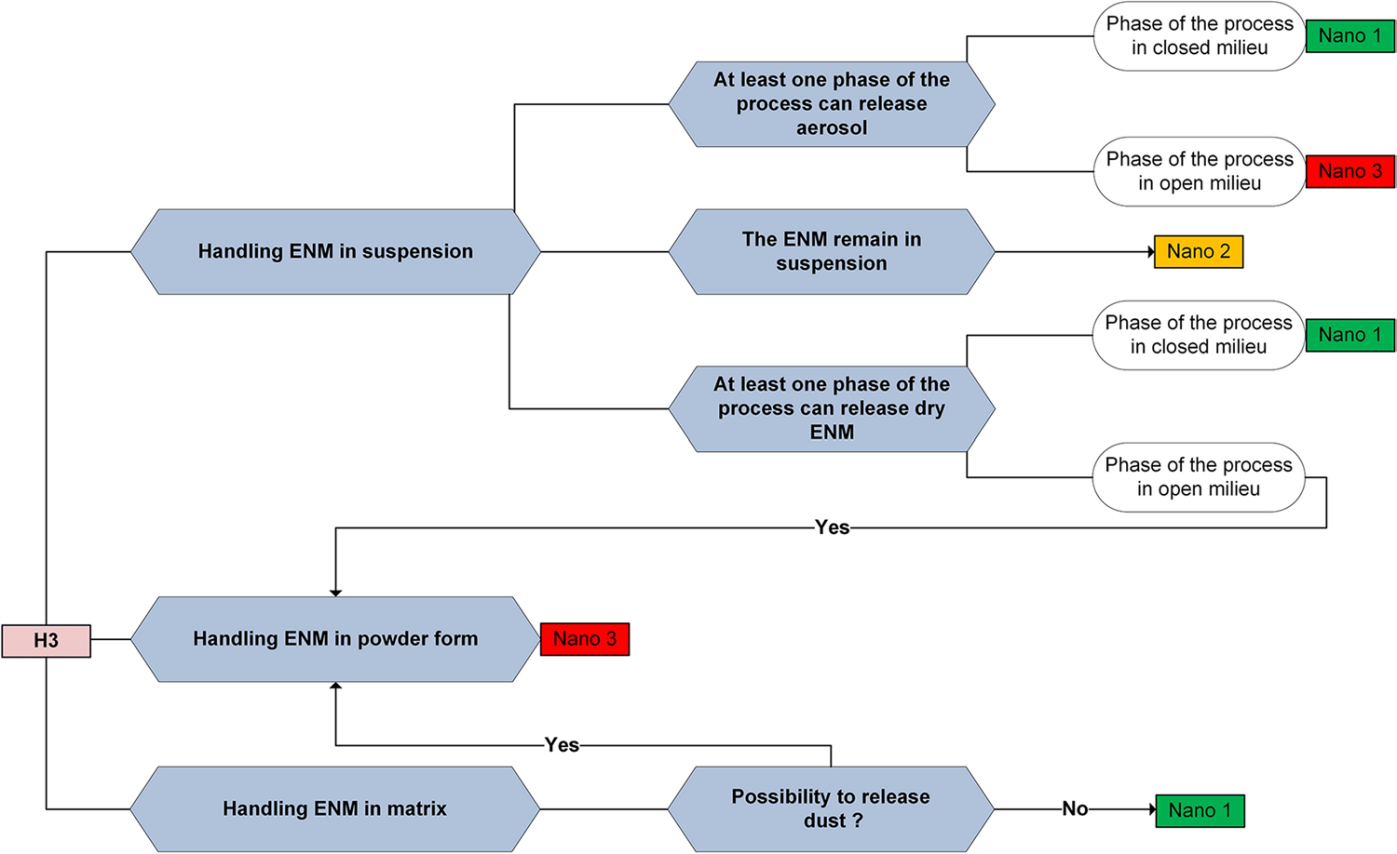
Decision tree for determining the Nano risk laboratory level. Decision tree used for determining the Nano level as a function of the substance and activity emission potential for the potential hazard level H3 obtained using Fig. 3

Once that one of the potential hazard levels (H1, H2, or H3) is assigned to the ENM, the substance and process emission potential is examined more in detail in the decision trees in Figs. 4, 5 in function of the obtained potential hazard level.

### Integration of the activity emission potential to obtain the nanosafety laboratory level

For the assigned potential hazard level (H1, H2, or H3), users should employ the corresponding decision tree (a, b, or c) in Figs. 4, 5 to determine the nanosafety laboratory level. The decision tree should be used to analyze each phase of the process (weighting, synthesis, etc.), as every stage represents a different activity emission potential. As a result of the analysis, the different phases of the processes will be classified into Nano 1, 2, or 3. The highest of the obtained nanosafety levels is then assigned to the laboratory (physical space).

#### Basis for the activity emission estimate

As discussed in the introduction, the risk of an event is determined as the product of the severity of the consequence of that event and its probability of occurrence. For substances that pose chronic hazards, the probability of appearance depends on the exposure dose to that substance and its efficiency to cause damage.

For ENMs, OELs and standard equipment for sufficiently detailed routine exposure measurements do not exist. Consequently, different alternative approaches are used to access the probability of occurrence and exposure. According modified (for ENM) source receptor approach [57], inhalation exposure is represented as a multiplicative function of the substance emission potential, activity emission potential, near field and far field sources, reduction of transmission (local controls and general ventilation), reduction of emission, and background [15]. When examining the maximum possible exposure, only the potential for emission of a process has been considered; the last being determined by the substance emission potential (form, physical state of the substance, dustiness) and the activity emission potential (handled amount and frequency of activity) [19]. For each of the decision trees that concern different hazard levels (Fig. 4a, b), we take the physical state of the substance (handling in suspension, powder, and matrix) as the starting point to assess the substance emission potential. The activity emission potential is then investigated only for powders, whereas, for handling of suspensions and matrices that remain in that phase, the activity emission potential is not investigated, as the substance emission potential is considered low.

*Handling ENMs in suspension.* If the ENMs remain in suspension, the laboratory is classified as Nano 1 (Fig. 4a, b); this classification is also applied for suspensions that are aerosolized in a closed environment. Only for H3 ENMs, even for activities where the ENMs remain in suspension, the attributed laboratory level is Nano 2 (Fig. 5). If the suspension is drying, it is treated as handling the ENM in powder form.

*Handling ENMs in powder form.* In case of H3 hazard level (Fig. 5), which is defined as the hazard level with significant or permanent health effects, it has been decided to classify the laboratory directly as Nano 3, independently of the quantities and frequency of use. The highly toxic aspect overwhelmed the frequency factor. For H1 and H2 potential hazard levels (Fig. 4a, b), the quantity of the powders as well as the frequency and duration of use are examined.

Concerning the choice of the relevant quantities of ENM powders to determine the activity emission potential, we noticed that typical amounts used in the literature [17, 58] are based on what is usually found in the environment, i.e., industry or research; it is difficult to determine some other reliable criteria for these quantities.

According to the guidelines on the precautionary matrix for synthetic nanomaterials developed by the Swiss Federal office of public health [59], the maximum possible exposure corresponds to the total quantity of the used substance. In the precautionary matrix, the threshold values for the quantities of the substance are calculated based on the OEL value for diesel soot particles. Starting from 100 μg/m^3^ (8 h weighted average limit concentration) and taking into account that average volume of air breathed by a 70 kg body weight person is 12 m^3^ in 8 h the obtained value is 1200 μg. The inhalation rate value assumed here is calculated using empirical formula Q_inh_ = 2.3 × B_w_^0.65^ m^3^/day for volume of air inhaled per hour by an adult in case of light exercise [60, 61]. If the entire quantity of the material is diffused in the air and inhaled, the value would still be below the limit value. In an equivalent manner, starting from the value of 3 mg/m^3^, a typical OEL for a multitude of inert particles [62] we have obtained a total quantity of 36 mg (rounded to 40 mg) for the inert materials. This value has been used as a threshold for materials whose hazard level is H1 (Fig. 4a). Three threshold values are used: quantities smaller than 40 mg (called Quantity 1), between 40 and 400 mg (Quantity 2), and larger than 400 mg (Quantity 3). A twice smaller quantity is used for H2 substances; the threshold values are organized in a similar way (20 mg, between 20 and 200 mg, and more than 200 mg) (Fig. 4b).

**Fig. 6:**
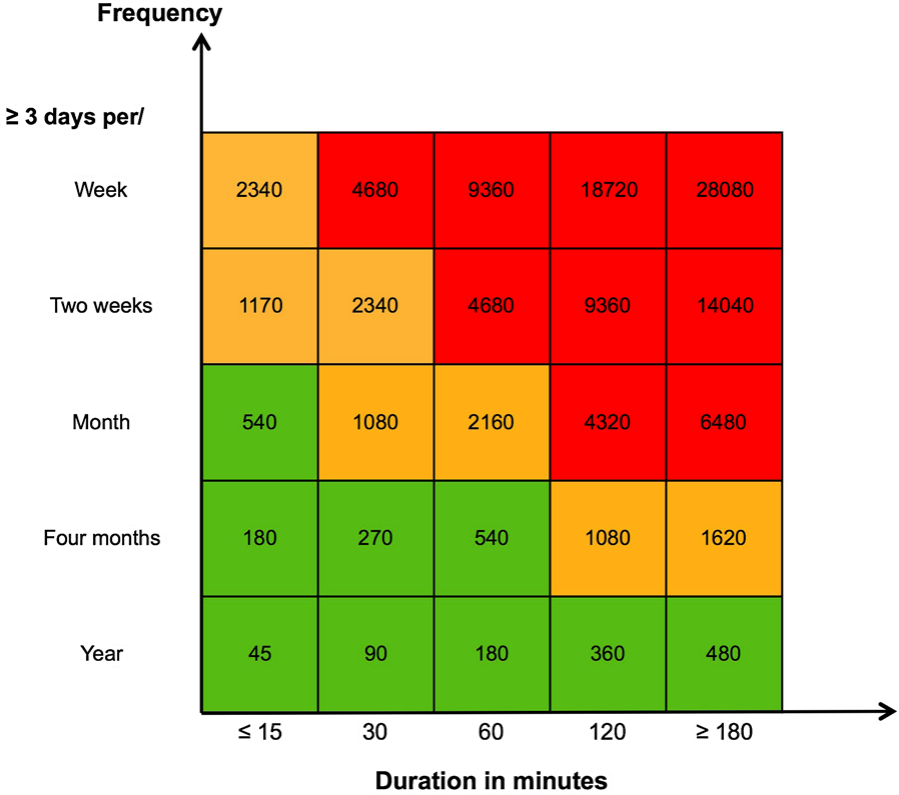
Matrix combining duration and frequency of operation for powder handling. Distribution of typical durations of operations in minutes (abscissa) in relation to the number of days of work in a year (ordinate). The numbers in the matrix are obtained as a product of the duration time (as a percentage of the 8 h working time) and number of days of work per year. Three classes of duration–frequency are set in *green*, *orange*, and *red*

We used the matrix presented in Fig. 6 to solve the frequency and duration of operation, which contribute to the activity emission potential together with the powder quantity. Typical durations of operations in research environments are presented on the abscissa (15, 30, 60, 120, and 180 min), while 3 days per week (ordinate) are considered as the average time that an individual works in the laboratory. Figure 6 shows the distribution of daily occupations in relation to the number of days of work in a year (1 week, 2 weeks, a month, 4 months and 52 weeks). Numbers in the matrix are obtained as the product of the activity duration time (expressed as the percentage of the 8 h working time) and the number of days of work per year. Three classes of frequency and duration are set in green, orange, and red.

The matrix used to correlate the quantities of the powders with the frequency and duration in the activity emission potential (EP 1, EP 2, and EP 3) is depicted in Additional file 1: Figure S2a. The matrix presenting the combinations used for finally combining H levels and EP levels into Nano laboratory levels is depicted in Additional file 1: Figure S2b.

In practice, the user does not need these two figures, as the matrices in there were already considered when designing the decision trees for the nanolevel determination (Figs. 4, 5).

*Handling ENMs in matrices.* The preparation of composites is either treated as handling ENMs in suspension or handling ENMs in powder. For all the potential hazard levels, if the material characterization and post-preparation processing activities do not include any mechanical or thermal treatment, the laboratory is classified as Nano 1 (Figs. 4, 5) in case of handling ENMs in matrix. If dust can be released during manipulations or if the composites are friable, the activity is treated as ‘handling ENMs in powder’.

### Protective and preventive measures

**Fig. 7:**
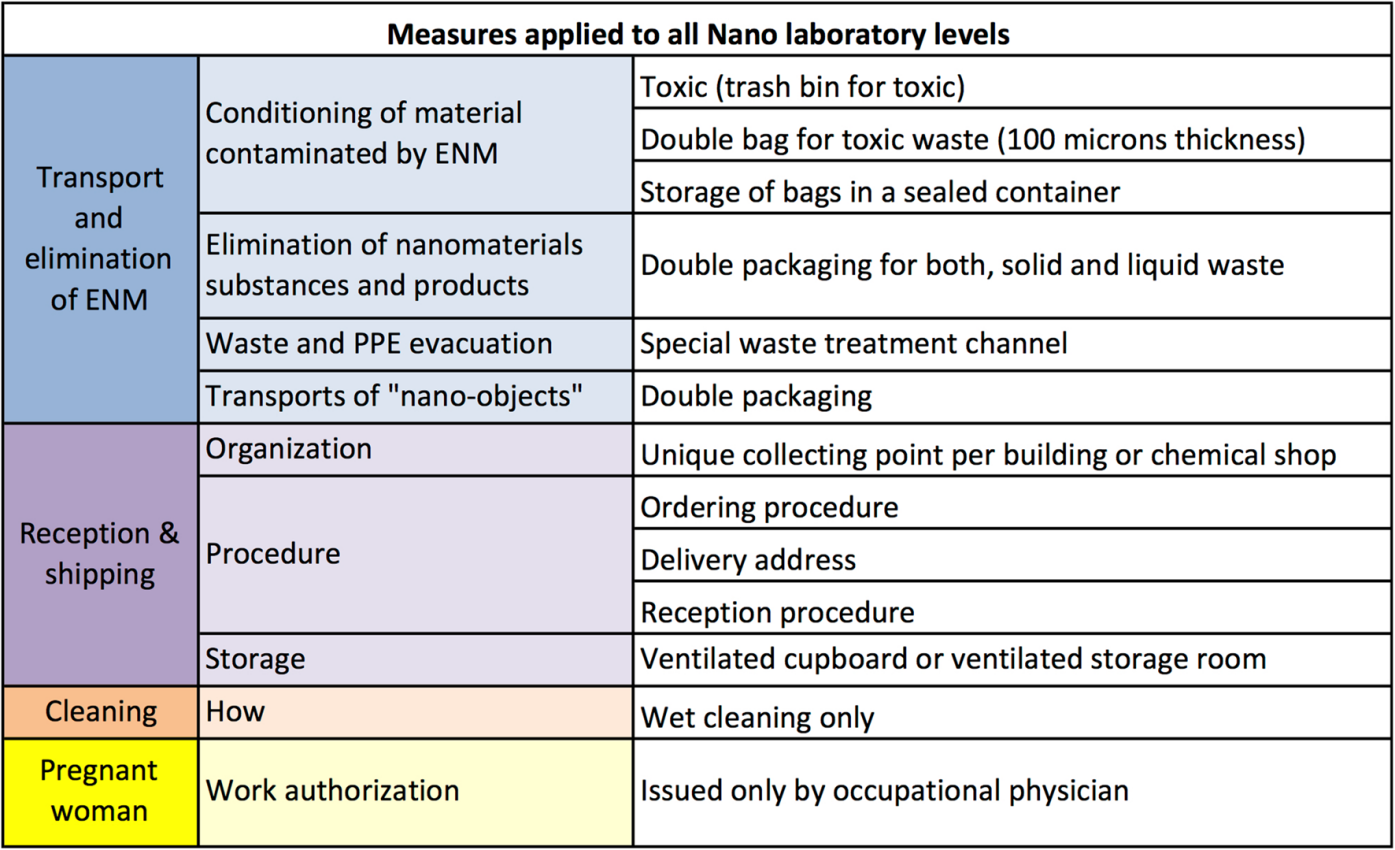
Protective measures for all the nanolaboratory types. These measures regard transport and elimination, reception, shipping, and cleaning

**Fig. 8:**
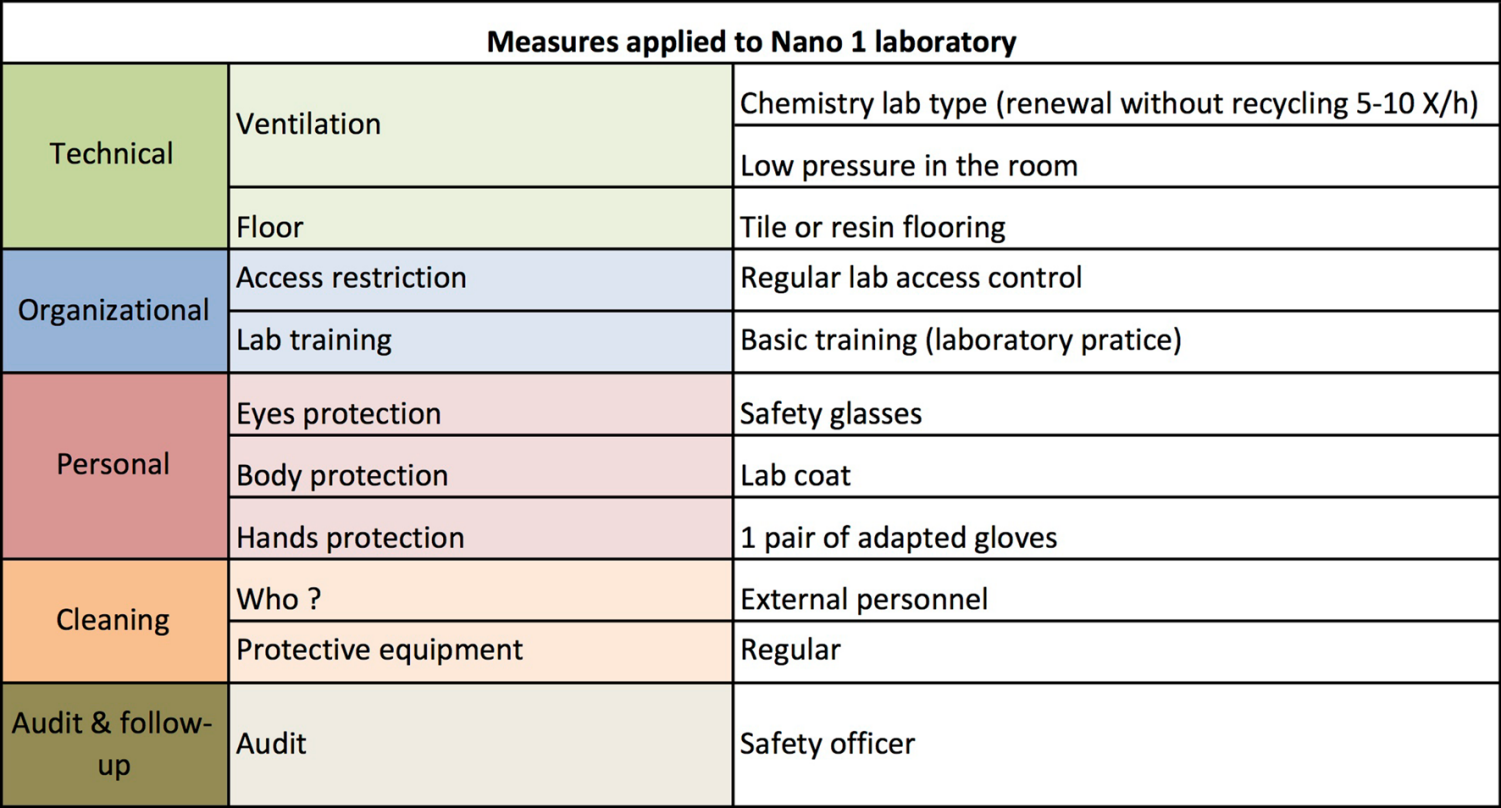
Protective measures for Nano 1 laboratories. The measures are organized into technical, organizational, and personal. The Nano 1 laboratory basically corresponds to an ordinary chemistry laboratory

**Fig. 9:**
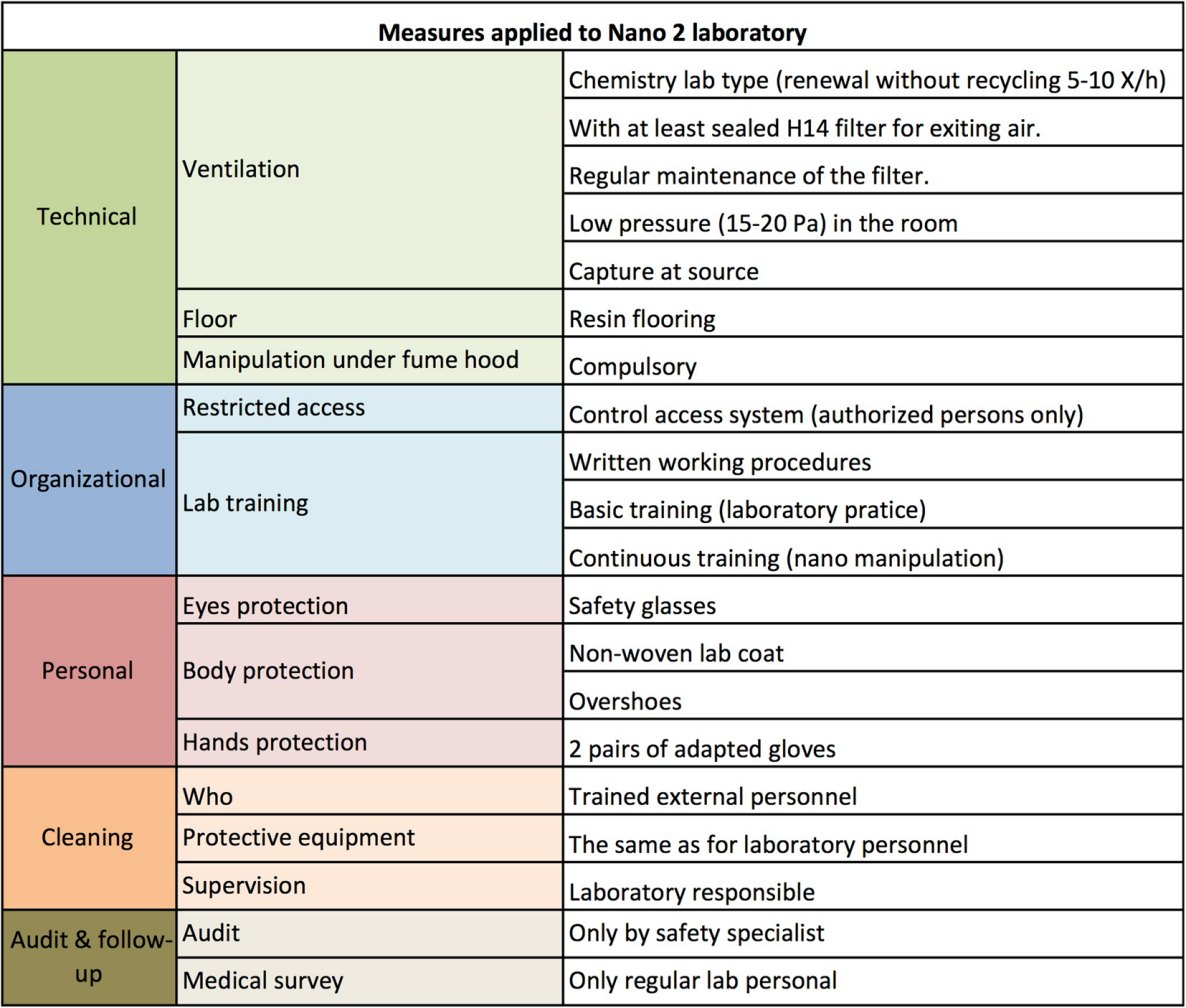
Protective measures for Nano 2 laboratories. The measures are organized into technical, organizational, and personal. Cleaning (person, protective equipment, and supervision) and medical survey are also specified
- Nano 3 laboratories (Fig. 10) require extensive technical measures. General exhaust ventilation should have exiting air filtering with a sealed H14 filter (EN 1822:2009 European Standard for ventilation filters. H14 has 99.995 % average efficiency for 0.3 μm particles) with regular maintenance. All the extraction air from a Nano 3 laboratory is filtered before reaching the ventilation system of the building to avoid air contamination outside the laboratory. Depending on the type of process and activity, either local exhaust ventilation, which captures contaminated emissions at or very near the source, or manipulation under chemical fume hood will be adopted.

**Fig. 10:**
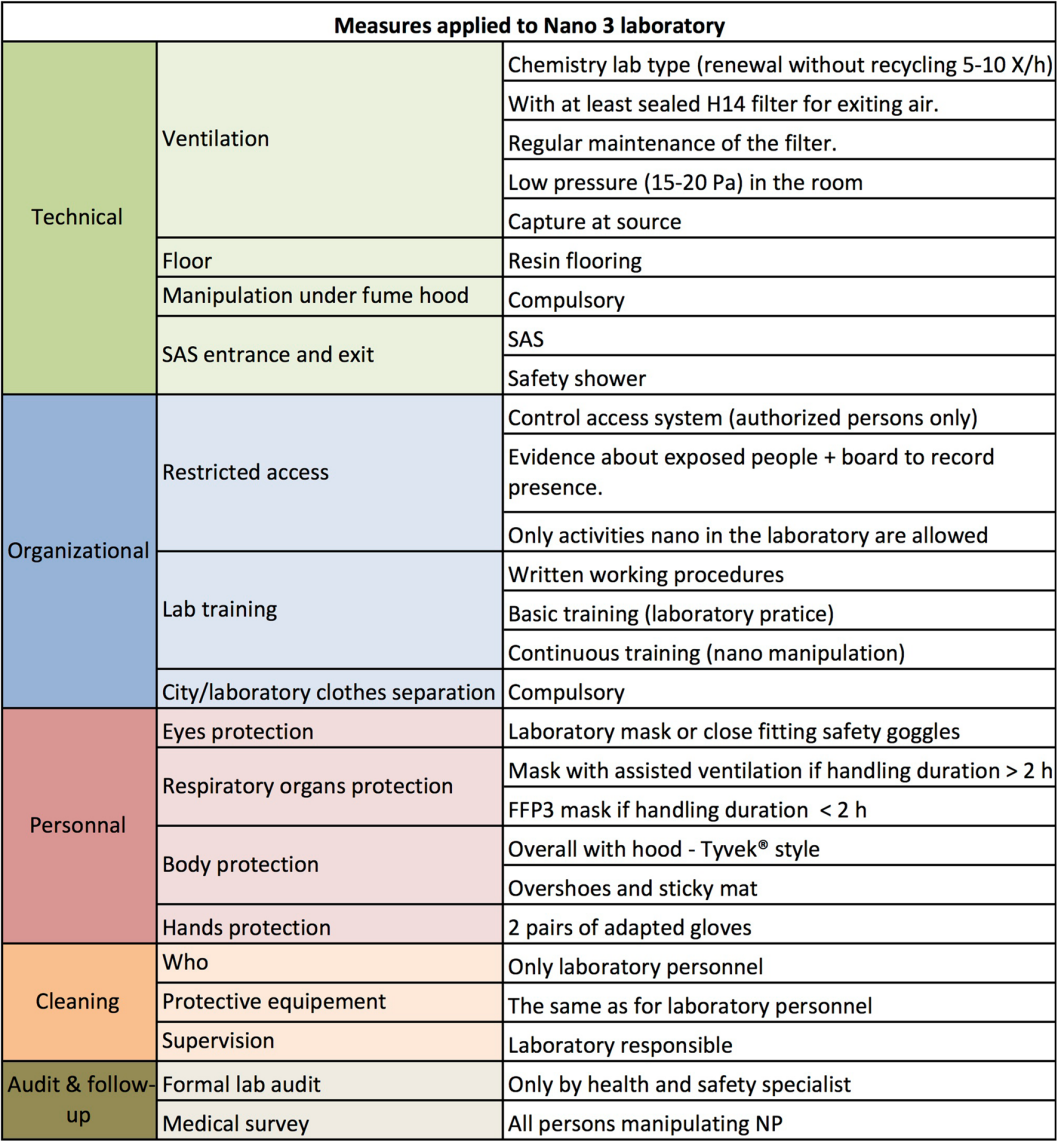
Protective measures for Nano 3 laboratories. The measures are organized into technical, organizational, and personal. Cleaning (person, protective equipment, and supervision) and medical survey are also specified
- Most of the organizational measures are present in all the Nano laboratory types as basic training on laboratory practice, introducing notions of ENM potential hazards, precautions, and written working procedures. Some additional organizational measures are recommended for the Nano 3 laboratory type, such as restricted access and laboratory training specific for the ENMs in use.
- A security vestibule with a safety shower (to use in case of accidents) is also required for Nano 3 laboratories. The former will be an area that is physically separated from the laboratory where clothes can be changed into laboratory apparel, preventing contamination of the former.
- Personal protective measures assign specific protective equipment to different hazard levels. As powered air respirator ensures better comfort for longer work periods, we recommend this system if the work (Nano 3) lasts over 2 h. While a P3 (EN 143) or FFP3 (EN 149)/P-100 (USA NIOSH) filter or filtering mask is advised for shorter work periods. Protection of the body parts depends on the hazard level, from laboratory coat for Nano 1 (Fig. 8) to overalls with hood (Tyvek^®^ style) for Nano 3 (Fig. 10). According to a work by Golanski et al. [63], the glove (nitrile, vinyl, latex, neoprene) efficiency is very high for 20–100 nm graphite aerosols. A recent study [64] showed penetration of nTiO_2_ for disposable nitrile gloves under deformation when exposed to both nTiO_2_ powder and colloidal solutions of nTiO_2_ (after 7 and 5 h respectively). The result suggests that the exposure might be reduced through frequent replacement of disposable gloves, especially for exposures to ENMs in liquid phase.
- We recommend the use of two pairs of gloves in case of Nano 3 and Nano 2 for precautionary purpose. By using two pairs of gloves, the user has the possibility to remove the contaminated pair of gloves first and keep the second pair until the protective clothes and mask are removed. One pair of gloves can be used for the Nano 1 laboratory level; choice of gloves must also take into account chemicals (i.e., solvents, surfactants, etc.) that might be part of the ENM matrix or use conditions.
- Currently, there are no national or international standards for medical survey. We recommend the medical survey (Figs. 9, 10) as a mandatory preventive examination for persons working with ENMs. Based on the Swiss National Insurance Company (SUVA) initiative, the survey is done (with 2 years interval) when two exclusive eligibility criteria are fulfilled:
- Work in areas classified as Nano 2 or Nano 3.
- Annual duration of exposure longer than 30 days or 200 h.

The preventive and protective measures that apply to the corresponding Nano safety laboratory level are summarized in Figs. 7, 8, 9, 10.

Mitigation measures that concern waste management, reception and shipment of ENMs, ordering procedures, and storage as well as cleaning (‘How’) are common for all the Nano laboratory safety levels and are regrouped in Fig. 7.

To reduce the ENM inhalation risk, it is strongly recommended to generate “liquid” waste. Contaminated material should be disposed in plastic bags for toxics placed inside trash bins. Solid waste should be disposed in adequate containers, one family of solids per container, and liquid waste in plastic containers, one type of solvent per container. Double packaging of containers should be used for the transport to the waste collecting point.

Besides the measures that are common to all the Nano laboratory safety levels, each level is characterized by a specific set of protective measures (Figs. 8, 9, 10) that are categorized into technical, organizational, and personal measures. The priority is set to protecting the respiratory tract and skin.

- As illustrated in Fig. 8, Nano 1 practically designates an ordinary chemistry laboratory with no particular requirements in terms of technical and personal protective measures.
- Nano 2 and Nano 3 laboratories basically have the same technical measures; Nano 2 laboratories (Fig. 9) are planned in such a way as to be technically ready to become Nano 3 laboratories. However, personal measures are considerably simpler.

Medical surveillance includes a targeted history, physical examination, laboratory testing (hematology, renal and hepatic parameters, urinary status), spirometry, electrocardiogram, and chest X-rays.

When downgrading a laboratory (from level 3 to 2, or from level 2 to 1), wet cleaning and double wiping should be done as well as contamination checks (air and surfaces) conducted by an occupational hygienist.

### Example of application of procedure for safety management of ENMs

#### Purification of silica particles

**Fig. 11:**
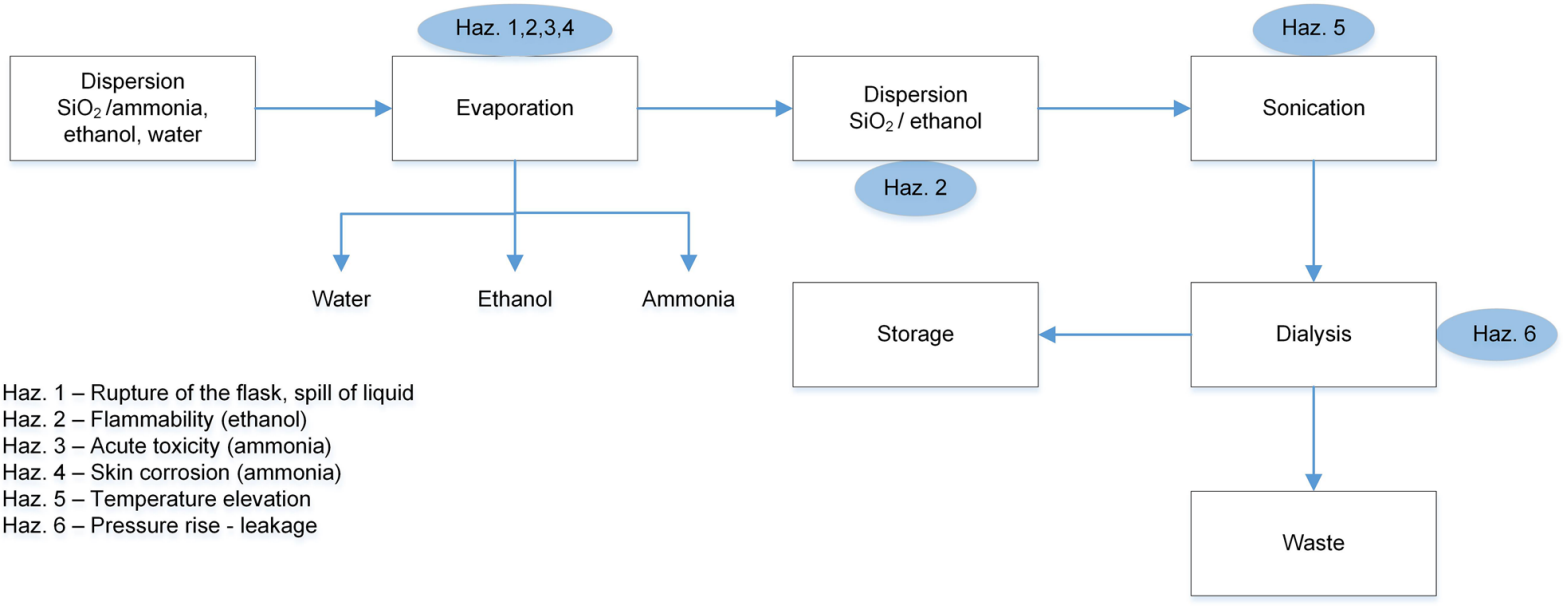
*Block diagram* representing the main steps of purification of silica particles. The *diagram* representing the main steps and related hazards (non ENM related) in the activity of purification of silica nanoparticles

*Description.* Typically, 250 mL of a silica particles dispersion is produced at approximately 10 g/L. This dispersion (in mixture of ethanol/ammonia/water) must be concentrated *in vacuo* prior to dialysis against water. The round bottom flask containing the particles is connected to a rotavap placed in a fume hood (pressure 250–300 mbar; temperature: 50 °C). The particles are subsequently transferred to a dialysis membrane (previously swollen in water). To transfer the particles adhered to the flask wall, a small volume of ethanol (5–10 mL) is added, and the flask is sonicated for 1 min. The liquid is then transferred to the dialysis membrane. Once the dialysis is complete, the particles (still in liquid) are stored in a bottle for further use. Final quantity: between 100 and 200 mL of dispersion; frequency of operation: few times per month. A block diagram in Fig. 11 represents the main steps of the activity and related (non related to ENM) hazards.

#### Evaluation using the safety procedure

The decision tree in Fig. 3 will be used to classify this material to one of the three potential hazard levels as follows.

- The process is not in a confined environment.
- Section *a* in Fig. 3: the relevant authority did not classify the ENM.
- Section *b*: bulk material does exist and is classified as non-hazardous. Further criteria are therefore examined in the following sections of the decision tree.
- Section *c*: the ENM is not soluble in water.
- Section *d*: the ENM is not a nanofiber.
- Section *e*: the material is not a pure metal or an alloy with metallic properties.
- Section *f*: the material is not pure C and is amorphous; As the substance is SiO_2_ it will be classified as H2.

*Results.* The exposure potential is examined using Fig. 4b, which relates to H2. As in all phases of the process the material remains in suspension, the process is classified as Nano 1.

The work can thus be done in an ordinary chemistry laboratory (Fig. 8, measures specific to Nano 1). Additional measures should be taken regarding the waste elimination and transport (Fig. 7).

Another example of application of procedure for safety management of ENMs (Handling of CNTs for cell culture experiment) is given in Additional file 2.

## Discussion

The testing in the field suggests the developed procedure is indeed simple and rapid to use analysis tool. The researchers can quickly assess the risks related to their activities with ENMs and identify which safety measures should be implemented. Based on new data on ENM, more differentiation is possible when classifying individual materials into hazard classes. The major asset of the preceding method regarding the level of details in preventive and protective measures in all its aspects is kept and further improved.

If the user did not invest sufficient time in examining the products or the working processes, thereby answering questions with ‘I do not know’, the laboratory will automatically be shifted to a higher Nano class. It is in the user’s interest to gather available information and answer the questions as accurately as possible.

When testing the procedure, the laboratory personnel would often skip a phase in the analysis of a process. As they are typically not trained for exposure risk analysis, they might oversee some segments where a product is transferred between containers or similar. The reminder that each phase of the work with ENMs should be studied is essential. In any case, the use of the presented procedure signifies an essential learning opportunity and contributes to the ENM safety awareness increase for the users, but also for employers and supervisors of ENM laboratories.

If a laboratory is classified as Nano 2 or Nano 3, the laboratory responsible will get in touch with the OHS specialist to analyze the process in more detail and consider the possibilities to reduce the Nano class or regrouping activities. If technically feasible, the process containment is the best option, as other protective measures are then avoided and the laboratory is not restricted to only certain nano–related activities. Following the analysis and expertise of the OHS specialists, the laboratory is finally classified. Protective measures should be taken as general recommendations; more complicated situations need to be individually analyzed and specific measures adopted. However, these specific analyses are restricted to a few specific circumstances, as the supplied tables cover the majority of the situations.

Concerning accidental releases, there is not a single procedure that can be suggested, as the reaction will depend on place where the spillage occurred, physical state and quantity of the substance in question. Small spillages of powder inside the enclosures can be wet cleaned. For liquid spills: absorbent material appropriate for the solvent in dispersion should be applied (all should then be eliminated to the special waste).

For spillage outside the enclosures, the staff should quit the laboratory and contact institute emergency service, which will isolate contaminated area and decide what action to take. For larger quantities of powder for example, a dedicated, approved HEPA vacuum whose filtration effectiveness has been verified can be used, and wet cleaning done subsequently.

As research activities, and therefore processes, change rapidly in research settings, it is crucial to quickly adapt to these changes. To fulfill this requirement, regular laboratory visits by occupational health and safety staff, information transfer to occupational safety and health staff, and updates of the nano evaluation whenever a notable change is observed in the activity classifications are mandatory. Implementation of presented procedure requires full support of parent organization. At EPFL, the school direction approved an internal directive on this subject.

## Conclusions

At present there is not enough knowledge on the possible adverse effects of ENMs to perform a complete quantitative risk assessment. This situation stimulates initiatives of governmental authorities, policy makers, industrial organizations, and civil society organizations to advocate the application of the precautionary principle for risk management.

Here, a practical and simple to use decision tree for nanomaterial safety and health management in research environments, based on the current state of knowledge, is proposed. It can be easily updated when more scientific data will become available.

The methodology is based on user-friendly questionnaires allowing the quantification of the ENM risk level of the laboratories to propose pragmatic mitigation measures and limit the exposures as much as considered reasonable. The laboratory responsible is in charge of applying the measures adapted to the specific activities. As research processes and equipment evolve very fast, the use of simplified methods with dedicated corrective measures and actions is essential. Monitoring, adaptation, control, and review of measures in place have a high importance.

Owing to an insufficient knowledge and application of the precautionary approach in practically all methodologies for management of ENM safety currently available, protective measures have an ‘over-protection’ tendency. There is a chance that these measures will be softened as knowledge will increase. As far as academia research is concerned, being on the ‘over-protective’ side is generally better than having an insufficient protection; this environment has an important role in educating people (they will be the future managers in the industry).

## Additional files

10.1186/s12951-016-0169-x **Figure S1** – Table with calculated values of band gaps and lower levels of the conductive band of some selected materials as functions of their particle size. **Figure S2** **a.** Matrix used to combine the quantities of powders with frequency and duration to obtain the activity emission potential (EP 1, EP 2, and EP 3). **b.** Matrix used for combining the potential H level with the EP level to obtain the Nano (1, 2, or 3) laboratory level.
10.1186/s12951-016-0169-x Additional example of application of procedure for safety management of ENMs (handling of CNTs for cell culture experiment).
