# Supplementary material for: Engineered nanomaterials: toward effective safety management in research laboratories
Source: J Nanobiotechnology. 2016 Mar 15;14:21. doi: 10.1186/s12951-016-0169-x (PMC4791936; doi:10.1186/s12951-016-0169-x)
Supplement: Supplementary file 1 — 10.1186/s12951-016-0169-x Figure S1 – Table with calculated values of band gaps and lower levels of the conductive band of some selected materials as functions of their particle size. Figure S2 a. Matrix used to combine the quantities of powders with frequency and duration to obtain the activity emission potential (EP 1, EP 2, and EP 3). b. Matrix used for combining the potential H level with the EP level to obtain the Nano (1, 2, or 3) laboratory level. [file 12951_2016_169_MOESM1_ESM.pdf]

Figure S1

Particles Size (nm)	Substance	Bandgap	Energy of lower level of conductance band
**5**	CuO_2	2.38	-4.84
	Alpha Fe_2O_3	2.23	-4.87
	Gamma Fe_2O_3	2.67	-4.69
	Fe_3O_4	2.09	-4.88
	WO_3	3.45	-5.33
	CoO	2.71	-4.27
	Mn_2O_3	3.23	-4.53
	Ni_2O_3	3.62	-4.19
	TiO_2 Anatase	4.09	-3.78
	TiO_2 Rutile	3.13	-4.52
	SnO_2 Rutile	4.25	-3.88
	CeO_2	3.89	-3.68
**10**	CuO_2	2.22	-4.91
	Alpha Fe_2O_3	2.05	-4.96
	Gamma Fe_2O_3	2.49	-4.78
	Fe_3O_4	1.91	-4.97
	WO_3	3.15	-5.48
	CoO	2.49	-4.38
	Mn_2O_3	3.05	-4.62
	Ni_2O_3	3.44	-4.28
	TiO_2 Anatase	3.52	-4.06
	TiO_2 Rutile	3.07	-4.55
	SnO_2 Rutile	4.06	-3.98
	CeO_2	3.71	-3.77
**25 - 100**	CuO_2	2.17	-4.94
	Alpha Fe_2O_3	1.99	-4.99
	Gamma Fe_2O_3	2.43	-4.80
	Fe_3O_4	1.85	-4.99
	WO_3	3.05	-5.53
	CoO	2.41	-4.42
	Mn_2O_3	2.99	-4.65
	Ni_2O_3	3.38	-4.31
	TiO_2 Anatase	3.33	-4.16
	TiO_2 Rutile	3.05	-4.56
	SnO_2 Rutile	4	-4.01
	CeO_2	3.65	-3.79

Figure S1 – Table with calculated values of band gaps and lower levels of the conductive band of some selected materials as functions of their particle size.

Figure S2

	Quantity 1	Quantity 2	Quantity 3
Frequency/ Duration Red	EP 2	EP 3	EP 3
Frequency/ Duration Orange	EP 1	EP 2	EP 3
Frequency/ Duration Green	EP 1	EP 1	EP 2

a

	H1	H2	H3
EP 3	Nano 3	Nano 3	Nano 3
EP 2	Nano 1	Nano 2	Nano 3
EP 1	Nano 1	Nano 1	Nano 2

b

Figure S2 a. Matrix used to combine the quantities of powders with frequency and duration to obtain the activity emission potential (EP 1, EP 2, and EP 3). **b.** Matrix used for combining the potential H level with the EP level to obtain the Nano (1, 2, or 3) laboratory level.
