# Supplementary material for: Engineered nanomaterials: toward effective safety management in research laboratories
Source: J Nanobiotechnology. 2016 Mar 15;14:21. doi: 10.1186/s12951-016-0169-x (PMC4791936; doi:10.1186/s12951-016-0169-x)
Supplement: Supplementary file 2 — 10.1186/s12951-016-0169-x Additional example of application of procedure for safety management of ENMs (handling of CNTs for cell culture experiment). [file 12951_2016_169_MOESM2_ESM.pdf]

Handling of CNTs for cell culture experiments

Description: CNTs are typically suspended from dry powder (weighted out at the milligrams stated, 1–4 mg per tube) in a fume hood as described in [1]. Cells are then exposed to the suspended CNT samples in a laminar flow hood within a biosafety level 2 cell culture laboratory. Cells are usually exposed for several hours, and then various assays are performed to assess the cell responses. In a recent study, five different types of CNTs were studied [2]: bundled single-walled CNT, bundled multi-walled CNTs, long, straight, stiff multi-walled CNTs, tangled multi-walled CNTs, and short and straight multi-walled CNTs as described in [1-4].

Frequency: this process is repeated once a week for 3.5 h.

Evaluation using the safety procedure. When analyzing the activities using our decision tree, the analyses concerning the different types of materials and different phases of the process are conducted separately; the highest obtained level has to be adopted for the laboratory. Out of the five studied CNT types, the multi-walled CNTs that are long (56 µm), straight, and stiff, according to Donaldson [3], are biopersistent. The decision tree in Figure 3 will be used to classify this material to one of the three potential hazard levels as follows.

- The process is not performed in a confined environment.
- Section **a** of Figure 3: the relevant authority did not classify the ENM.
- Section **b**: bulk material does not exist.
- Section **c**: the ENM is not soluble.
- Section **d**: the long multi-walled CNT is a biopersistent nanofiber; it is therefore classified as potential hazard level H3.

Results. To estimate the process exposure potential, the weighting of the multi-walled CNT in a dry form is analyzed first. Using Figure 4c (that concerns H3) will yield

Nano 3, independently of the frequency of use and quantity of product. Other parts of the process that involve a suspension phase will classify the laboratory as Nano 2. Preferably, the activity assigning the highest Nano level to the laboratory should be enclosed (powder handling enclosure for example [5]). If that is not possible, the laboratory as all will be classified as Nano 3 (since the highest of the obtained levels should be taken in the end). It will therefore be equipped according to the measures cited in Figure 6 (general measures for all the Nano laboratories) and Figure 9 (specific for Nano 3).

References

1. Wick P, Manser P, Limbach LK, Dettlaff-Weglikowska U, Krumeich F, Roth S et al. The degree and kind of agglomeration affect carbon nanotube cytotoxicity. Toxicol Lett. 2007;168(2):121-31. doi:10.1016/j.toxlet.2006.08.019.
2. Clift MJD, Frey S, Endes C, Hirsch V, Kuhn DA, Johnston BD et al. Assessing the impact of the physical properties of industrially produced carbon nanotubes on their interaction with human primary macrophages in vitro. BioNanoMat 2013;14(3-4):239-48.
3. Poland CA, Duffin R, Kinloch I, Maynard A, Wallace WAH, Seaton A et al. Carbon nanotubes introduced into the abdominal cavity of mice show asbestos-like pathogenicity in a pilot study. Nat Nanotechnol. 2008;3(7):423-28. doi:10.1038/nnano.2008.111.
4. Thurnherr T, Su DS, Diener L, Weinberg G, Manser P, Pfaender N et al. Comprehensive evaluation of in vitro toxicity of three large-scale produced carbon nanotubes on human Jurkat T cells and a comparison to crocidolite asbestos. Nanotoxicology. 2009;3(4):319-38. doi:10.3109/17435390903276958.
5. Departement of health and human services NIOSH. General Safe Practices for Working with Engineered Nanomaterials in Research Laboratories 2012. Report No.: 2012-147.
